# Effect of Calcination Temperature of FeCoO_x_/Al_2_O_3_ Catalyst on the Catalytic Pyrolysis of High-Density Polyethylene

**DOI:** 10.3390/ma19112340

**Published:** 2026-06-01

**Authors:** Xuemei Zheng, Ying Zhang, Xulong Yang, Chao Yuwen, Bingguo Liu, Aiyuan Ma

**Affiliations:** 1 School of Metallurgical and Energy Engineering, Kunming University of Science and Technology, Kunming 650093, China; zxm_lpssy19@163.com (X.Z.);; 2 Key Laboratory of Unconventional Metallurgy, Ministry of Education, School of Metallurgical and Energy Engineering, Kunming University of Science and Technology, Kunming 650093, China; 3 National Local Joint Laboratory of Engineering Application of Microwave Energy and Equipment Technology, Kunming University of Science and Technology, Kunming 650093, China; 4 School of Chemistry and Materials Engineering, Liupanshui Normal University, Liupanshui 553004, China; zy2000202109@163.com (Y.Z.);

**Keywords:** high-density polyethylene, FeCoO_x_/Al_2_O_3_ catalyst, plastic pyrolysis, calcination temperature

## Abstract

Catalytic pyrolysis has emerged as a promising approach for converting waste plastics into high-value-added chemicals and fuels. This study aims to investigate the effect of calcination temperature on the catalytic performance of FeCoO_x_/Al_2_O_3_ catalysts for high-density polyethylene (HDPE) pyrolysis and to optimize the catalyst preparation conditions for maximizing valuable product yields. FeCoO_x_/Al_2_O_3_ catalysts were synthesized via a hydrothermal method and calcined at various temperatures (300–700 °C). The results demonstrate that calcination temperature significantly influences product distribution: gas yield increased with rising calcination temperature, whereas carbon yield, hydrogen yield, and hydrogen content decreased accordingly. Among all tested temperatures, the catalyst calcined at 500 °C achieved the optimal performance, yielding solid carbon at 23.0 wt. % with a hydrogen content of 80 vol.%. This superior performance can be attributed to its larger specific surface area, a richer pore structure, and better reducibility compared to those calcined at higher temperatures, which also facilitated the formation of solid carbon with the highest degree of graphitization and purity. This work provides technical guidance for the high-value utilization of waste plastics through catalytic pyrolysis.

## 1. Introduction

Plastics have been essential materials in modern society due to their low cost, durability, ease of processing, light weight, and chemical resistance [1,2]. Unfortunately, just because of their non-biodegradability, combined with their extensive use, large amounts of plastic-derived waste has posed a serious threat to the global environment and biosecurity [1,2,3]. According to statistics [4], over 1.2 billion tons of plastic waste was generated globally in the past three years, yet only about 9% were recycled, whereas most of the remainder was disposed of by deep burial or incineration and discharged into natural environment heavily, causing air, soil and water pollution and increasing the risk of exposure to microplastics [5]. Therefore, it is an urgent issue to deal with waste plastics more effectively and cleanly from the perspective of sustainable development. Currently, catalytic pyrolysis has attracted much attention because it can convert waste plastics into high-value-added chemicals such as hydrocarbons, aldehydes, alcohols, syngas, fuel oil, high-quality carbon materials, etc. [6,7,8,9,10]. Furthermore, as an important medium in the plastic pyrolysis process, the design and synthesis of catalysts are at the core of upgrading plastic pyrolysis [11,12].

Transition metals, especially iron (Fe), nickel (Ni), and cobalt (Co), were commonly used in the catalytic pyrolysis of waste plastics due to their low cost and catalytic efficiency [13,14,15]. In addition, alumina (Al_2_O_3_), possessing good thermal stability and suitable acidic sites, was often used as a catalyst support to load active components and disperse active metal components [16,17]. In previous studies, Acomb [18] used catalysts of different transition metals (Ni, Fe, Co, Cu) loaded on alumina (Al_2_O_3_) by impregnation to pyrolyze Low-Density Polyethylene (LDPE); the final distribution of plastic pyrolysis products varied significantly due to the differences in the strength of the interaction between the active components and the Al_2_O_3_ carrier; Yao et al. [19] loaded Fe-Ni bimetallic catalysts on Al_2_O_3_ and found that Fe-Ni bimetallic-based catalysts had stable performance, good metal dispersion, and more appropriate interaction with the carrier, compared with Fe/Al_2_O_3_ and Ni/Al_2_O_3_ catalytic pyrolysis; Liu et al. [20] loaded Ni, Co and bimetallic Ni-Co on Al_2_O_3_ to catalyze the pyrolysis of polyethylene and the bimetallic catalyst showed higher pyrolysis activity and achieved a carbon yield of 25.50 wt. % due to the interaction between the Ni/Co bimetallic and the Al_2_O_3_ phase. However, it has been found from existing studies that the catalytic performance of supported catalysts mainly depends on the selection of active metal elements and the composition of active components, and the structural composition and texture characteristics of the catalysts mainly depend on the heat treatment process in the preparation process, among which the calcination temperature directly determines the crystal phase structure, grain size and specific surface area of the catalyst [21,22]. It also significantly affects the interaction between active components and the support, the distribution of acidic sites on the surface, and the redox properties of the metal oxides [17,23]. Among the numerous catalysts, FeCo-based catalysts have attracted much attention due to their low cost and availability, excellent C-C bond breaking ability, and their unique electronic structure and synergistic catalytic effect of bimetallic oxides [24,25]. For example, for Fe-based and Co-based catalysts, changing calcination temperature may induce the formation or decomposition of spinel phases (such as CoFe_2_O_4_) and alter the occupancy of cations in the lattice, thereby regulating the catalytic active centers [26,27]. In areas such as syngas conversion, NH_3_-SCR, and CO oxidation, there have been many studies on the influence of structural and physical properties of FeCoO_x_/Al_2_O_3_ catalyst regulated by adjusting the calcination temperature. However, the specific role of calcination temperature in tailoring the structural evolution and catalytic properties of the FeCoO_x_/Al_2_O_3_ catalyst, and its subsequent impact on plastic pyrolysis performance and product selectivity, has not been systematically elucidated [28,29].

In this study, FeCoOx/Al_2_O_3_ catalysts were synthesized by hydrothermal treatment to address the unclear mechanisms by which the structural properties of FeCoO_x_/Al_2_O_3_ catalysts were affected by calcination temperature and the unclear structure–activity relationship for high-density polyethylene (HDPE) pyrolysis. The crystallinity and texture properties were regulated by changing the calcination temperature, and the dispersion morphology of active species was optimized. The in situ catalytic pyrolysis of HDPE was conducted at a fixed catalytic temperature (500 °C), and the three-phase product yield, gas composition, and the morphology, structure and quality of solid carbon were systematically analyzed to clarify the effect of calcination temperature on the plastic pyrolysis performance and product selectivity of the FeCoO_x_/Al_2_O_3_ catalyst. The results of this study can provide a theoretical basis for the design of efficient and stable plastic pyrolysis catalysts and technical support for the resource utilization of waste plastics.

## 2. Experimental Section

### 2.1. Materials

High-density polyethylene (HDPE) was commercially sourced, with elemental contents of C 86.47 wt.%, H 13.45 wt.%, and S 0.07 wt.%, which were analyzed by an elemental analyzer (Varig EL.Cube, Germany). Additionally, the moisture content of 0.04 wt.%, volatile content of 99.79 wt.% and ash content of 0.17 wt.% in HDPE were also measured in accordance with ASTM standards E790, E897 and E830 [12], respectively, as shown in Table 1.

Cobalt nitrate hexahydrate (Co(NO_3_)_2_⋅6H_2_O, analytical-grade), iron nitrate nonahydrate (Fe(NO)·_3_9H_2_O, analytical grade)_3_, aluminum oxide (Al_2_O_3_, analytical-grade), and anhydrous ethanol (C_2_H_5_OH, 99%) used in the experiment were all purchased from Tianjin Zhiyuan Chemical Reagent Co., Ltd. (Tianjin, China). Urea (CH_4_N_2_O, 99%) and aluminum sec-butanol (ATB) were purchased from Maclean reagent and Cia reagent, respectively, and the water used in the experiment was deionized water made in the laboratory.

### 2.2. Catalyst Synthesis

The catalysts used in this paper were prepared by hydrothermal synthesis. Firstly, Fe(NO_3_)_3_·9H_2_O, Co(NO_3_)_2_·6H_2_O and urea (CH_4_N_2_O) were dissolved in 50 mL of deionized water according to a molar ratio of 1:2:3 and stirred for 30 min to fully dissolve and react to form a suspension. Afterwards, 1 g of Al_2_O_3_ was gradually added into the suspension, and the pH of the mixture was adjusted to 9.0 by dropping ammonia solution (13.3 mol/L), and the stirring was performed for 1 h to form a stable brown flocculent suspension. The suspension was transferred into a 100 mL stainless steel autoclave and maintained at 140 °C for 12 h. After cooling to room temperature, the solid product was collected by filtration, washed with deionized water until neutral, and then vacuum-dried at 100 °C for 12 h to obtain the precursor. Finally, the precursor was calcined in a muffle furnace at a rate of 10 °C/min to 500 °C, 600 °C, 700 °C, or 800 °C for 3 h; the target catalysts were obtained and named “FeCoO_x_/Al_2_O_3_-T, T = 500, 600, 700, 800”, respectively.

### 2.3. Experimental Setup and Procedure

**Figure 1:**
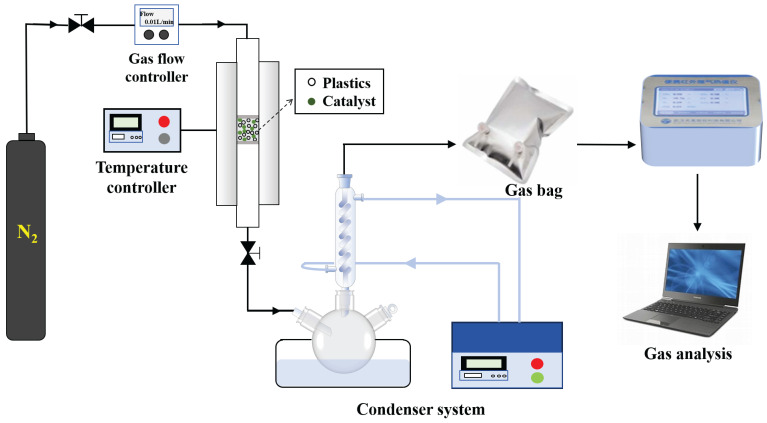
Schematic diagram of pyrolysis experimental setup.

The pyrolysis experimental setup is shown in Figure 1, including the gas supply system, pyrolysis reactor, condensation unit, gas collection and analytical detection system.

Specifically, the catalytic pyrolysis process was as follows: Firstly, 1.8 g of HDPE powder and 3.6 g of catalyst were ground and loaded into a quartz boat, then placed in the center of the tube. Subsequently, nitrogen was purged into the reaction system at a flow rate of 200 mL/min for 20 min, followed by heating the sample to 500 °C at 10 °C/min and holding for 1 h. After the pyrolysis was completed, the system was purged with nitrogen at a flow rate of 100 mL/min for 30 min, and the residual pyrolysis gas in the reactor was vented into the gas collection bag. The gas was quantitatively analyzed using a portable infrared gas analyzer (TY-6330P, Wuhan Tianyu Intelligent Control Technology Co., Ltd., Wuhan, China).

After the pyrolysis process was completed, the masses of the quartz boat, tube and condensers were weighed to calculate the yields of the liquid, solid and gas. According to Zhao et al.’s study [30], the liquid yield, solid yield, gas yield and hydrogen yield were calculated by Equations (1), (2), (3) and (4), respectively. In order to ensure the reliability of the data, each experiment was repeated three times, and the average value was taken as the final result.

(1) $$\mathrm{Liquid}\;\mathrm{yield}\;(\mathrm{wt}.\;\%)=\frac{M_{2}-M_{1}}{M_{\mathrm{plastics}}}\;\times \;100\%$$

(2) $$\mathrm{Solid}\;\mathrm{yield}\;(\mathrm{wt}.\;\%)=\frac{M_{\mathrm{tot}}-M_{\mathrm{cat}}+M_{O}}{M_{\mathrm{plastics}}}\;\times \;100\%$$

(3) Gas yield (wt. %)=100−Liquid yield−Solid yield

(4) $$H_{2}(\mathrm{mmol}\cdot g_{\mathrm{plastics}}^{-1})=\frac{\mathrm{Mole}\;\mathrm{of}\;H_{2}\;\mathrm{produced}}{\mathrm{Mass}\;\mathrm{of}\;\mathrm{plastics}}$$

where M_1_ is the mass of quartz tube and the condensers before the experiment, g, M_2_ (g) is the mass of quartz tube and the condensers after the experiment, and M_plastic_ (g) is the mass of feed HDPE, M_tot_ (g) is the total mass of the catalyst and the solid product after the experiment, M_cat_ (g) is the mass of the catalyst, and M_O_ is the mass of oxygen consumed by the catalyst.

The Co-O/Fe-O bond strength is evaluated by Equation (5):

(5) $$\omega =\frac{1}{2\pi c}\sqrt{\frac{k}{\mu }}$$

where *ω* is the wavenumber (cm^−1^), *c* is the speed of light (m/s), *k* is the force constant of Co-O/Fe-O (N/m), and *μ* is the effective mass of the Co-O/Fe-O bond (g).

### 2.4. Characterization Methods

The N_2_ adsorption–desorption test of the catalyst sample was performed on a physical adsorption analyzer (ASAP 2020 PLUS, Micromeritics, USA) to analyze the specific surface area and pore structure. The catalyst was degassed under vacuum at 300 °C for 7 h. The specific surface area was calculated using the Brunauer–Emmett–Teller (BET) method, and the pore size distribution was determined by the Barrett–Joyner–Halenda (BJH) method based on the desorption isotherm data. X-ray photoelectron spectroscopy (XPS, Thermo Scientific K-Alpha, Waltham, MA, USA) was employed to analyze the elemental composition and chemical states of the fresh catalyst. The obtained XPS spectra provided information on the surface elemental composition, chemical states, and molecular structure based on the peak positions and shapes, while the surface elemental contents were derived from the peak intensities. Hydrogen temperature-programmed reduction (H_2_-TPR) was carried out on a chemisorption analyzer (TP-5080, China) to evaluate the redox properties of the catalyst. A 50 mg sample was loaded into a U-shaped quartz tube. The reduction was performed under an atmosphere of 5 vol.% H_2_ balanced with 95 vol.% N_2_, with the temperature ramped from room temperature to 800 °C at a rate of 10 °C·min^−1^. The signal was recorded using a thermal conductivity detector (TCD). X-ray diffraction (XRD, Rigaku Ultima IV, Japan) was utilized to analyze the phase composition, crystal structure, and interlayer spacing of both the fresh catalyst and the reaction products. The measurement conditions were as follows: Cu Kα radiation, tube voltage of 50 kV, tube current of 30 mA, scanning speed of 2°/min, and scanning range of 10–80°. Raman spectroscopy (Renishaw inVia, Germany) was performed on both the fresh and spent catalysts using a 532 nm excitation laser to analyze the changes in the Co–O/Fe–O bond vibrations of the fresh catalyst, as well as the degree of defects and graphitization of the solid carbon produced. Scanning electron microscopy (SEM, ZEISS Sigma 300, Germany) was used to observe the micromorphology of the fresh catalyst and the solid carbon. For sample preparation, a powder sample was attached to conductive tape, sputter-coated with gold, and then imaged at an accelerating voltage of 10 kV. Transmission electron microscopy (TEM, JEOL JEM-2100F, Japan) was further employed to examine the internal microstructure of the solid carbon product. The detailed procedure was as follows: a small amount of the solid carbon sample was dispersed in anhydrous ethanol, followed by ultrasonic dispersion for 5 min to achieve uniform dispersion. A disposable pipette was used to drop the suspension onto a carbon-coated copper TEM grid. After wicking away excess liquid with filter paper, the grid was placed into the sample holder. The observation was carried out under high vacuum at an operating voltage of 200 kV.

## 3. Results and Discussion

### 3.1. Catalyst Properties

**Figure 2:**
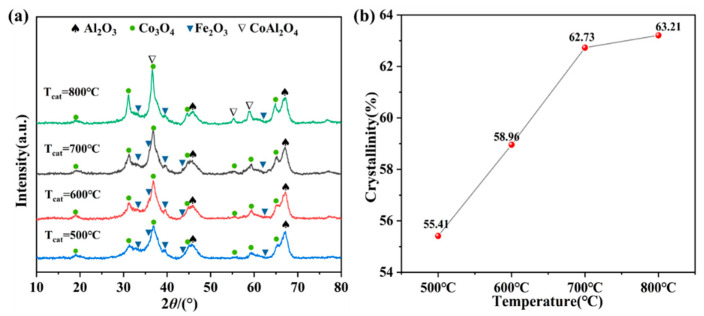
XRD patterns (**a**) and XRD crystallinity curves (**b**) of FeCoOx/Al_2_O_3_ catalysts prepared at different calcination temperatures.

The XRD patterns of catalysts prepared at different calcination temperatures are shown in Figure 2a. Apparently, distinct characteristic peaks corresponding to Co_3_O_4_ (PDF: 76-1802) were observed in all fresh catalysts at 19.0°, 31.3°, 36.9°, 44.8°, 55.7°, 59.4°, and 65.3°, assigned to the (111), (220), (311), (400), (422), (511), and (440) crystal planes, respectively. The peaks at 33.8°, 35.6°, 38.8°, 43.3°, and 62.9° correspond to α-Fe_2_O_3_ (PDF: 39-1346), indexed to the (310), (311), (320), (400), and (440) planes. The characteristic peaks at approximately 37.6°, 45.2°, and 67.1° correspond to the (311), (400), and (440) crystal planes of cubic Al_2_O_3_ (PDF: 29-0063). When the calcination temperature was 800 °C, additional characteristic peaks corresponding to the CoAl_2_O_4_ phase (PDF: 82-2245) appeared at 36.7°, 55.5°, and 59.2°, assigned to the (311), (422), and (511) crystal planes, which indicated that higher calcination temperatures might promote the migration and diffusion of cobalt, thereby facilitating the contact between cobalt and the active sites on the surface of the carrier, forming the CoAl_2_O_4_ spinel phase and altering the cation occupancy in the lattice, contributing to the regulation of the catalytic active center [26,27]. Furthermore, the crystallinity of fresh catalysts prepared at different calcination temperatures was calculated by XRD following the method described by Nara and Komiya, as shown in Figure 2b [31]. It can be seen that the crystallinity of catalysts improves with the rise in calcination temperature and has a higher crystallinity at 800 °C. This can be attributed to the significant increase in atomic migration rate in the catalyst, the continuous aggregation and growth of crystal nuclei, the gradual enhancement of Co^2+^ octahedral occupancy, the exponential rise in lattice reconstruction rate, and the significant increase in crystallinity [32].

**Figure 3:**
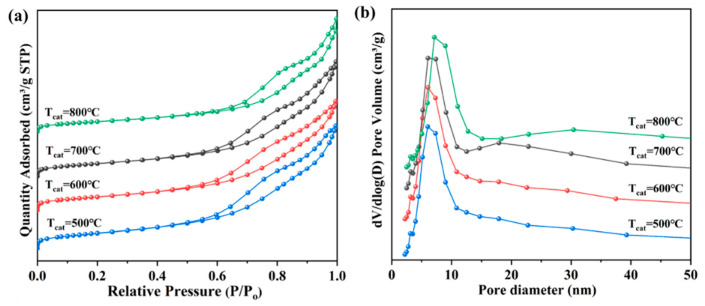
N_2_ adsorption–desorption isotherms (**a**) and pore size distribution plots (**b**) of FeCoOx/Al_2_O_3_ catalysts prepared at different calcination temperatures.

As shown in Figure 3, the N_2_ adsorption–desorption isotherms (a) and pore size distributions (b) of catalysts demonstrate that all catalysts prepared have a mesoporous structure and are consistent with Type IV, according to the classification of isothermal adsorption and desorption curves by IUPAC. In addition, detailed data on specific surface area, pore volume, and pore size of the catalysts are presented in Table 2. Specific surface area was calculated using the BET method, while pore size distribution was determined by the BJH procedure based on desorption isotherm data. The average pore size was mainly distributed in the range of 8.40–11.00 nm, and increased with the increase in calcination temperature, while the specific surface area and pore volume of the catalysts decreased inversely. It has been widely reported that excessively high calcination temperatures may induce partial sintering and structural collapse of catalysts, thereby reducing their catalytic activity [33]. In light of this, the catalytic performance of the FeCoO_x_/Al_2_O_3_ catalysts prepared at different calcination temperatures will be systematically evaluated and discussed in the following section.

**Figure 4:**
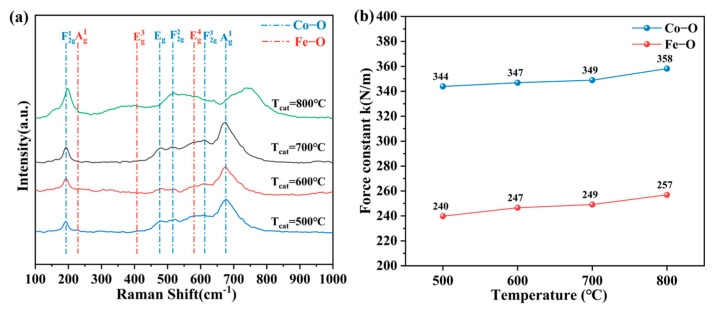
Raman spectra (**a**) and variation curves of Fe-O and Co-O force constants (**b**) for FeCoO_x_/Al_2_O_3_ catalysts prepared at different calcination temperatures.

In the Raman spectra of each sample in Figure 4a, synthesized catalysts showed clear stretching vibrations at approximately 190 cm^−1^, 480 cm^−1^, 520 cm^−1^, 620 cm^−1^ and 690 cm^−1^ corresponding to the $F_{2g}^{1}$, $E_{g},$ $F_{2g}^{2}$, $F_{2g}^{3}$ and $A_{g}^{1}$ modes of Co_3_O_4_ and relatively weak stretching vibration at approximately 225 cm^−1^, 410 cm^−1^, and 590 cm^−1^ corresponding to the $A_{g}^{1}$, $F_{2g}^{3}$ and $F_{2g}^{4}$ modes of Fe_2_O_3_ [34]. Furthermore, the intensities of the Co-O/Fe-O in the 680–690 cm^−1^ and 580–590 cm^−1^ vibration peaks were calculated respectively by Hooke’s law [35] (Equation (5)), as shown in Figure 4b. The results show that with the increase in calcination temperature, the force constant of the Co-O/Fe-O bond in the catalyst gradually increased. Particularly, at the calcination temperature of 800 °C, FeCoO_x_/Al_2_O_3_ appeared to have a significant blue shift, and it was observed that the $A_{g}^{1}$ characteristic peak of Co_3_O_4_ was significantly weakened while a strong stretching vibration appeared at 750 cm^−1^. This might be due to the higher temperature promoting the dispersion of Co, which had a strong interaction with the Al_2_O_3_ support to form the CoAl_2_O_4_ spinel phase [36], consistent with the XRD analysis results. Yet the catalyst with a calcination temperature of 500 °C had a rich specific surface area and porosity, a weaker Co-O/Fe-O strength, as well as preferring bond breaking and reduction, and thus had higher catalytic activity.

**Figure 5:**
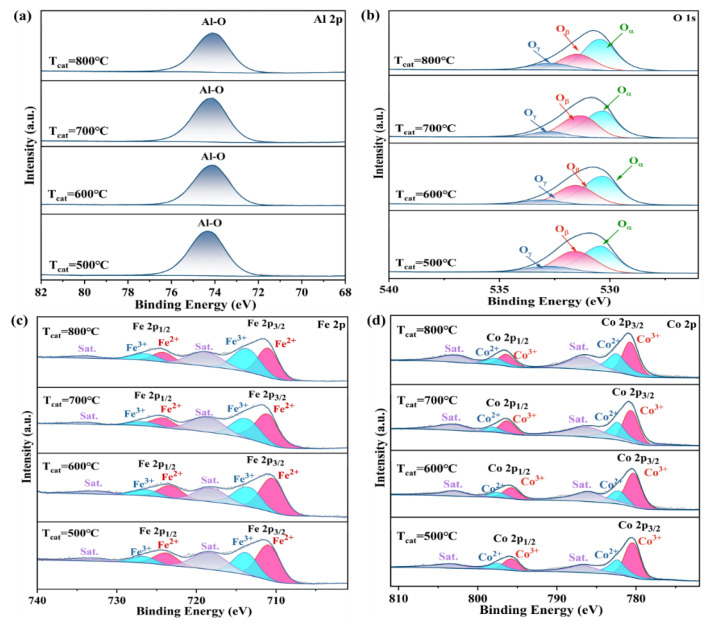
XPS characterization of FeCoO_x_/Al_2_O_3_ catalysts prepared at different calcination temperatures: (**a**) Al 2p; (**b**) O 1s; (**c**) Fe 2p; (**d**) Co 2p.

The XPS spectra for Al 2p (a), O 1s (b), Fe 2p (c) and Co 2p (d) are presented in Figure 5. The Al 2p spectra of the four catalysts showed a peak at approximately 74.0 eV, confirming the presence of the Al-O bond in the catalysts [37,38]. The Fe 2p (c) and Co 2p (d) signals in Figure 5c,d reveal that the metals on the catalyst surface exist in their Fe^2+^/Co^2+^ and Fe^3+^/Co^3+^ oxidation states. The O 1s spectrum of the catalyst exhibited three distinct peaks, namely lattice oxygen O**_α_** (529.1–530.0 eV), chemically adsorbed oxygen O**_β_** (531.0 eV), and hydrated adsorbed oxygen O**_γ_** (532.2 eV). O**_β_** reflects oxygen vacancies, and the higher the proportion of O**_β_**, the higher the catalytic activity of the catalyst. In addition, Table 3 shows the content of O on the surface of the catalysts and the Fe^2+^/Fe^3+^ and Co^2+^/Co^3+^ ratios calculated based on peak area. The results indicated that O**_β_** gradually decreased as the calcination temperature increased, which can be attributed to the fact that higher temperature promotes the growth of catalyst crystals and the reduction in defects. As the calcination temperature rose, the Fe^2+^/Fe^3+^ ratio gradually decreased while the Co^2+^/Co^3+^ ratio did the opposite, indicating that Fe^3+^ and Co^3+^ in the catalyst were gradually increasing, possibly because the increase in O**_α_** led to an increase in the mobility of oxygen ions in the metal oxide lattice, promoting the transformation from Fe^2+^ to Fe^3+^. The increase in Co^2+^ is due to the fact that at high temperatures, Co^2+^ diffuses into Al_2_O_3_, forming spinel CoAl_2_O_4_ [39].

**Figure 6:**
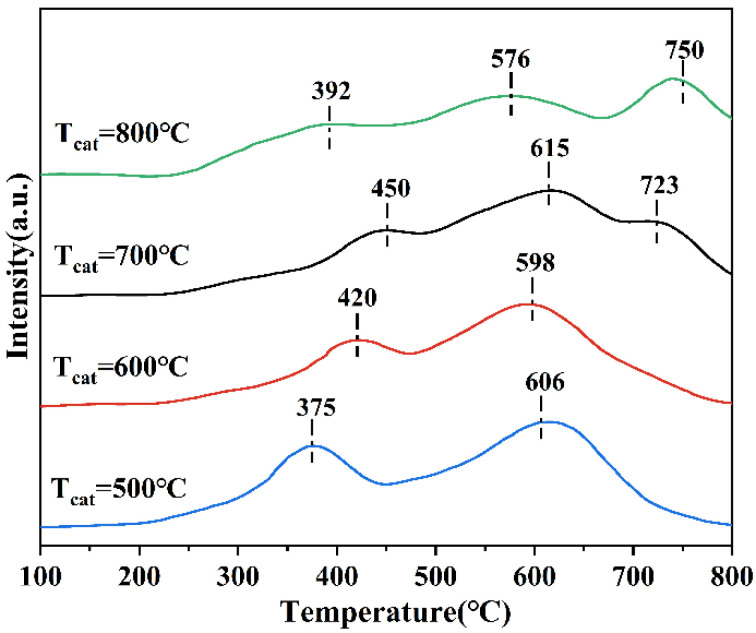
H_2_-TPR profiles of fresh catalysts prepared at different calcination temperatures.

To investigate the influence of calcination temperature on the reducibility of catalysts, the H_2_-TPR technique was adopted, and the result is shown in Figure 6. When calcination temperatures were 500 °C and 600 °C, the first reduction peak appeared at 375 °C–420 °C, corresponding to the reduction of Co_3_O_4_ or Fe_2_O_3_ to Co or Fe_3_O_4_ [40], and the second reduction peak, present at around 600 °C, stood for the reduction from magnetic Fe_3_O_4_ to unstable FeO and elemental Fe^0^, which was mainly caused by H_2_. However, at higher calcination temperatures, when they were 700 °C and 800 °C, a third distinct reduction peak was observed. This meant that the reduction of Fe_2_O_3_ in catalysts went through three stages: Fe_2_O_3_ was reduced to magnetic Fe_3_O_4_ at 450 °C/392 °C, from magnetic Fe_3_O_4_ to unstable FeO at approximately 615 °C/576 °C, and finally from unstable FeO to elemental Fe at 723 °C/750 °C. It was found that the reduction temperature is closely related to the metal–carrier interaction: the higher the reduction temperature, the stronger the interaction between the metal-active material and the carrier [41]. Thus, as the calcination temperature rises, the reduction temperature of the catalyst gradually moves towards higher temperatures, indicating that the interaction between the metal and the carrier is gradually strengthening.

### 3.2. Product Distribution and Gas Composition

**Figure 7:**
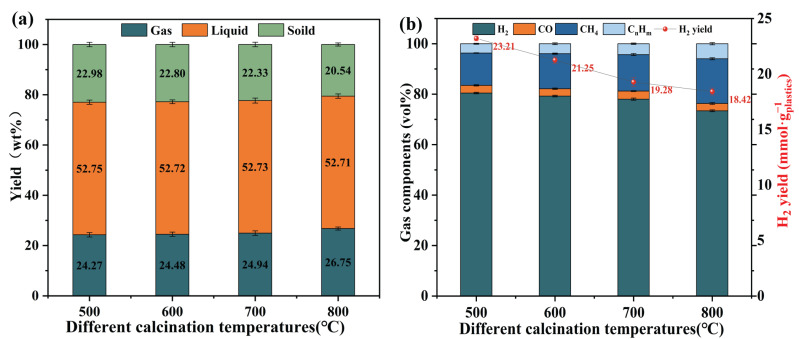
Product yield (**a**) and gas component distribution (**b**) of catalytic pyrolysis of HDPE.

Based on the above results, the performance of the catalyst varies depending on the preparation conditions, and different properties ultimately affect the product distribution and selectivity of catalytic pyrolysis. The product distribution of HDPE catalyzed by FeCoO_x_/Al_2_O_3_ prepared at different calcination temperatures is shown in Figure 7a. For catalysts prepared at different calcination temperatures from 500 °C to 800 °C, it seemed to have little effect on the yield of catalytic pyrolysis oil, while the gas yield gradually increased and solid yield gradually decreased, with the increase in calcination temperature, which is also the main focus of this study. To better understand the change, the gas component was analyzed, as presented in Figure 7b. Among them, H_2_ content was the highest, and gradually declined when the calcination temperature went up, while CH_4_ and C_n_H_m_ gradually increased. Particularly, higher solid carbon (23.0 wt.%) and hydrogen production (23.2 ${mmol\cdot g}_{\mathrm{plastics}}^{-1}$) were obtained at a calcination temperature of 500 °C. This further illustrates that the catalyst calcined at 500 °C has a larger specific surface area and pore structure to provide more active sites, thus promoting the deep dehydrogenation reaction between C_n_H_m_ generated from plastic chain breaking and metal active sites, and accelerating the dissolution of generated carbon atoms into the interior of metal nanoparticles, generating a large amount of deposited carbon. Conversely, the catalyst calcined at 800 °C has a smaller specific surface area, pore volume, and a larger force constant of the Co-O/Fe-O bond, making it difficult to break the bond and be reduced. So, the catalyst with lower catalytic activity is not conducive to the dehydrogenation of macromolecular hydrocarbons, resulting in the lowest hydrogen yield and solid carbon yield.

### 3.3. Carbon Products Analysis

**Figure 8:**
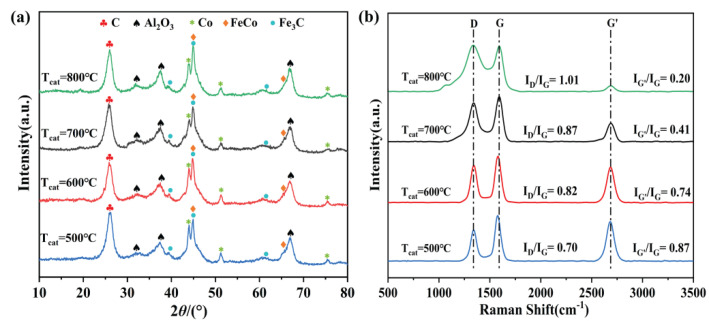
XRD (**a**) and Raman (**b**) spectra of pyrolytic solid products at different calcination temperatures.

Figure 8a depicts the XRD patterns for the spent catalysts. The typical (002) crystal plane (PDF: 75-1621) diffraction peaks of carbon material were detected near 26°, indicating the formation of graphite carbon. Fe_2_O_3_ in the fresh catalyst was mainly in the form of Fe_3_C (PDF: 35-0722) after reaction, indicating that Fe was involved in the process of carbon dissolution, while Co_3_O_4_ was present as elemental Co (PDF: 01-1259). More specifically, Co metal showed its (111) crystal peak at 44.2°, (200) crystal peak at 51.5°, and (211) crystal peak at 75.4°, while Fe_3_C peaks were present at 39.8°, 44.9°, and 61.4°, with crystal planes (002), (031), and (141), respectively. In addition, FeCo peaks (JCPDS No. 65-4131) were also detected at 44.8° and 65.3°, corresponding to the (110) and (200) crystal planes, indicating that hydrogen gas and reducing gas released during pyrolysis facilitated the partial reduction of iron cobalt oxide in the fresh catalyst to FeCo.

The Raman spectrum was utilized to detect the graphitization and purity of carbon products, as shown in Figure 8b. Characteristic Raman peaks near 1345 cm^−1^, 1573 cm^−1^ and 2680 cm^−1^ correspond to peaks D, G and G′, respectively. Peak D is formed by the vibration absorption mode of the six-membered ring activated by the edges of defects, amorphous carbon, or lamellar crystal surfaces, peak G is the Raman characteristic peak corresponding to the stretching vibration of C-C bonds between graphite lamellae of sp^2^-hybrid carbon atoms, and Peak G′ is produced by the secondary scattering process of two phonons. Generally, I_D_/I_G_, representing the intensity ratio of peak D to peak G, is used to calculate the graphitization extent and crystallization index, and high graphitization is matched with a small ratio [42], while the I_G′_/I_G_ ratio, representing the intensity ratio of peak G′ to peak G, indicates the purity of the carbon nanotube; the higher the ratio, the higher the purity. With the increase in calcination temperature, the degree of graphitization and purity of the carbon products gradually decreased and the defects on the graphite layer increased. When the calcination temperature was 500 °C, the I_G′_/I_G_ value was the smallest and the I/I value was the largest. This might be because the catalyst with a lower calcination temperature had a relatively large specific surface area and pore structure, providing abundant active sites, and the catalyst had moderate crystallinity, wide and weak M-O bonds, which were more conducive to the dispersion and reduction of metal oxides, promoting the dissolution of carbon atoms into the interior of metal nanoparticles after reaching supersaturation, resulting in large amounts of carbon nanotubes precipitating from the other side of the particle.

**Figure 9:**
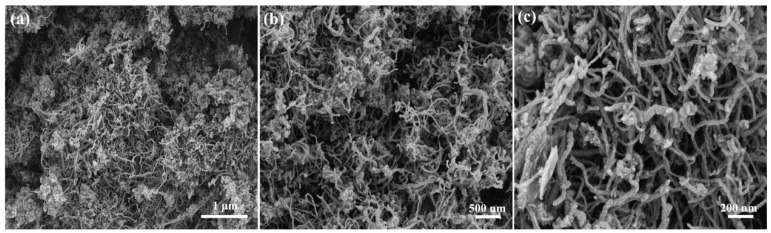
SEM images of carbon deposits on the catalyst calcined at 500 °C (**a**–**c**).

The morphology and structure of carbon products at the calcination temperature of 500 °C were further analyzed by scanning electron microscopy, as shown in Figure 9. Obviously, large amounts of fluffy filamentous carbon were covered on the surface of the catalyst, as well as intertwined with each other, confirming the presence of CNTs (carbon nanotubes).

**Figure 10:**
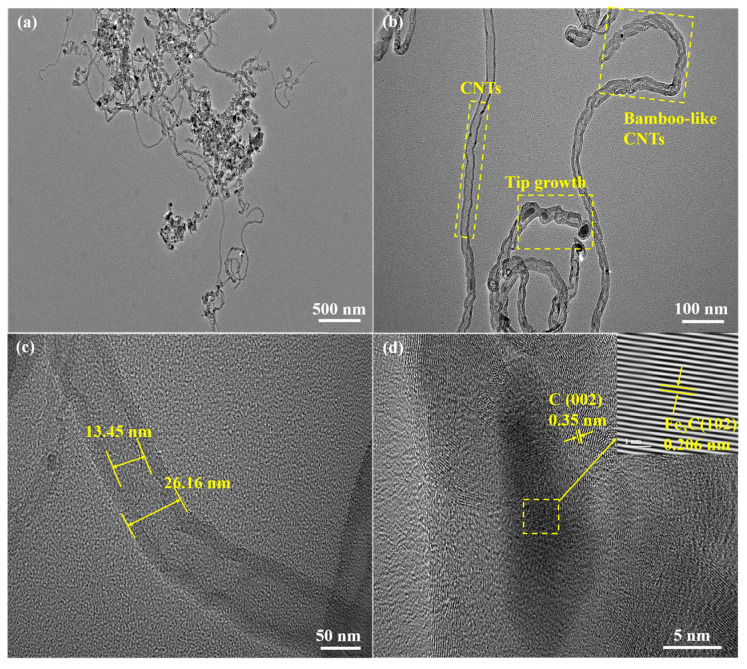
TEM image of CNTs: (**a**) Morphology of CNTs; (**b**) Growth mechanism and tubular structure; (**c**) Diameter of CNTs; (**d**) Active sites.

Additionally, the morphology and crystal structure of the deposited carbon were analyzed using TEM to further explore the growth mechanism of CNTs, as shown in Figure 10. From Figure 10, both filamentous carbon and amorphous carbon can be observed (a), and CNTs were mainly bamboo-like (b). Moreover, some metal nanoparticles of the catalyst were encapsulated at the tip or in the middle of nanotubes, providing a substrate for the growth of carbon nanotubes. In general, tip-growth and base-growth are prevailing descriptions for the CNT growth mechanism, in accordance with the location of nanoparticles inside the formed nanotubes [43]. Thus, the growth of CNTs in this study mainly follows the tip-growth mechanism [44]. As shown in Figure 10c, the generated carbon nanotubes had an outer diameter of 26.16 nm and an inner diameter of 13.45 nm, which meant hollow CNTs with thinner walls, and the carbon layer took on clear lattice fringes; the lattice spacing of the carbon layer was 0.35 nm, corresponding to the (002) crystal plane of graphite carbon, as shown in Figure 10d. As for the catalyst, clear lattice fringes were observed as well, and the lattice spacing was 0.206 nm, corresponding to the (102) crystal plane of Fe_3_C. This indicated that the carbon atoms in the pyrolysis products were dissolved within the metal nanoparticles and formed Fe_3_C, consistent with the results of XRD. Furthermore, when the carbon atoms reached saturation in nanoparticles, they continuously segregated and grew along the metal nanoparticles to form CNTs.

## 4. Conclusions

In this study, the influence of calcination temperature on the catalytic performance of FeCoO_x_/Al_2_O_3_ catalysts and the product distribution during catalytic pyrolysis of HDPE was systematically investigated. The results showed that lower calcination temperature improved the reducibility of the catalyst, whereas higher calcination temperature facilitated its crystallinity. From the perspective of product distribution, calcination temperature had little effect on the liquid proportion, and the gas proportion did not vary much, but as the pyrolysis temperature rose, the contents of C_n_H_m_ increased accordingly, which meant that the total of H_2_, CH_4_ and CO decreased gradually. When the calcination temperature was 500 °C, the hydrogen yield reached the maximum value of 23.21 ${mmol\cdot g}_{\mathrm{plastics}}^{-1}$, and the proportion of carbon products reached a higher value of 22.98 wt. %. Through further analysis of the carbon products, it was found that the degree of graphitization and purity of the solid carbon products gradually decreased with the increase in calcination temperature. At the calcination temperature of 500 °C, the degree of graphitization and purity of the carbon product were optimal, and filamentous carbon exhibited on the surface of the used catalyst mainly confirmed the presence of CNTs, according to the results of SEM and TEM. These findings provide fundamental insights into the structure–performance relationship of bimetallic catalysts in plastic catalytic pyrolysis. Nevertheless, catalyst recovery, CNT separation, and systematic benchmarking against other catalytic systems remain to be addressed in future work to further evaluate the practical applicability of this approach.

## Figures and Tables

**Table 1:** Elemental composition and industrial analysis of high-density polyethylene (HDPE).

Plastic	Elemental Analysis (wt.%)				Proximate Analysis (wt.%)		
	C	H	S	N	Moisture	Volatiles	Ash
HDPE	86.47	13.45	0.07	0.00	0.04	99.79	0.17

**Table 2:** Specific surface area, pore volume, and pore size distribution of FeCoOx/Al_2_O_3_ catalysts prepared at different calcination temperatures.

Catalyst	Surface Area (m^2^/g)	Pore Volume (mL/g)	Average Pore Size (nm)
FeCoO_x_/Al_2_O_3_-500 °C	124.2	0.4	8.4
FeCoO_x_/Al_2_O_3_-600 °C	111.4	0.3	8.4
FeCoO_x_/Al_2_O_3_-700 °C	106.6	0.3	9.3
FeCoO_x_/Al_2_O_3_-800 °C	88.6	0.3	11.2

**Table 3:** Surface atomic ratio data of fresh catalysts prepared at different calcination temperatures.

Catalyst	O_α_/O_all_	O_β_/O_all_	Fe^2+^/Fe^3+^	Co^2+^/Co^3+^
	%	%	Ratio	Ratio
FeCoO_x_/Al_2_O_3_-500 °C	46.52	39.33	1.63	0.61
FeCoO_x_/Al_2_O_3_-600 °C	48.70	35.97	1.42	0.74
FeCoO_x_/Al_2_O_3_-700 °C	51.82	34.11	1.35	0.81
FeCoO_x_/Al_2_O_3_-800 °C	52.80	32.22	0.91	1.00

## Data Availability

The original contributions presented in this study are included in the article. Further inquiries can be directed to the corresponding authors.
